# Influence of cutting time interval and season on productivity, nutrient partitioning, and forage quality of blue panicgrass (*Panicum antidotale* Retz.) under saline irrigation in Southern region of Morocco

**DOI:** 10.3389/fpls.2023.1186036

**Published:** 2023-06-07

**Authors:** Ayoub El Mouttaqi, Ihssane Mnaouer, Abdelaziz Nilahyane, Dennis S. Ashilenje, Erick Amombo, Mohamed Belcaid, Mohamed Ibourki, Karima Lazaar, Aziz Soulaimani, Krishna Prasad Devkota, Lamfeddal Kouisni, Abdelaziz Hirich

**Affiliations:** ^1^ Agriculture in Marginal Environment Program, African Sustainable Agriculture Research Institute (ASARI), Mohammed VI Polytechnic University (UM6P), Laayoune, Morocco; ^2^ Agricultural Innovation and Technology Transfer Center (AITTC), Mohammed VI Polytechnic University (UM6P), Ben Guerir, Morocco; ^3^ Soil, Water, and Agronomy (SWA) Program, International Center for Agricultural Research in the Dry Areas (ICARDA), Rabat, Morocco

**Keywords:** salinity, irrigation, forage yield, sodium, dry matter, protein

## Abstract

Salinity has become a major issue in various parts of the world negatively impacting agricultural activities and leading to diminished crop potential and lower yields. Such situation calls for urgent interventions such as adopting salt-tolerant crops to fill the gap in food and feed availability. Blue panicgrass (*Panicum antidotale* Retz.) is a promising salt-tolerant forage crop that has shown an appropriate adaptation and performance in the saline, arid, and desertic environments of southern Morocco. However, for obtaining a highest forage productivity with nutritional quality, optimization of the cutting interval is required. Thus, the objective of this study was to determine the optimal cutting time interval allowing high forage production and quality under high salinity conditions. This experiment was conducted over one entire year covering the summer and winter seasons. The effect of five cutting time intervals on selected agro-morphological traits, crop productivity, mineral nutrient accumulation, and forage quality of blue panicgrass in the region of Laayoune, southern Morocco. The finding of this study recommend that cutting blue panicgrass every 40 days maximized the annual fresh and dry forage yield as well as the protein yield, which reached 74, 22, and 2.9 t/ha, respectively. This study also revealed a significant effect of the season on both productivity and quality. However, forage yield declined during the winter and increased during the summer, while protein content increased during winter compared to summer. The mineral nutrient partitioning between shoots and roots, especially the K^+^/Na^+^ ratio, indicated that blue panicgrass has salt tolerance mechanism as it excluded sodium from the roots and compartmentalized it in the leaves. In conclusion, there is a potential of blue panicgrass on sustaining forage production under salt-affected drylands, as demonstrated by the response to two key questions: (a) a technical question to farmers for its adoption such as at which interval should blue panicgrass be harvested maximizing both forage yield and quality? And (b) a scientific question on how does blue panicgrass maintain high K^+^/Na^+^ ratio to cope with salinity stress?

## 1 Introduction

Agricultural production is limited by several factors such as impact of climate change, drought, desertification, and salinity. Soil salinity affected more than 424 million hectares of topsoil (0-30 cm) and 833 million hectares of subsoil (30-100 cm) (FAO, 2022). The adverse extent of salinity has attracted urgent interventions to adopt management practices and crops that can exploit salt-affected soil and water resources for economic value.

Livestock is a major component of the agricultural production system and is inextricably tied to forage production. This sector contributes to nearly 40 and 20% of the total agricultural output of developed and developing countries, respectively. Furthermore, it supports the livelihoods of more than 1.3 billion people worldwide (FAO, 2018). Those figures are likely to increase due to rapid demographic growth and urbanization, especially in the face of increasing effect of climate change and rainfall variability. To sustain livestock production, farmers need to improve forage production especially through the introduction of drought- and salt-tolerant and resilient forage crop species and providing site- and soil-specific customized good agronomic practice. One of the prudent solutions to achieve this goal is to adopt alternative forage crops which are more resilient to different stresses. These are crops with superior traits and resilience hence their introduction to a new environment overcomes the productivity challenges of traditional crops vulnerable to abiotic or biotic stresses (Elouafi et al., 2020). In this context, alternative crops provide a basket of choice to the farmers and might improve resilience through its tolerance to different stresses.

The adoption of salt-tolerant alternative forage crops is a key solution not only to overcome salinity problem but also to fill the gap in fodder availability and scarcity in marginal areas. Morocco is an excellent illustration of the influence of climate change-related drought on feed supply. In fact, as Verner et al. (2018) predict, drought in Morocco is increasing in frequency and intensity and this trend will likely be more intense in the future. In the most recent season (2021-2022), Morocco faced the worst effect of drought in three decades which led to feeding shortage and higher prices. To respond such effect and increase forage productivity, several salt-tolerant alternative crops have been introduced and tested under saline conditions in the south of Morocco (Hirich et al., 2021). A few of those introduced alternative crops such as quinoa (*Chenopodium quinoa* Willd.), sesbania (*Sesbania sesban* (L.) Merr.), blue panicgrass (*Panicum antidotale* Retz.), barley (*Hordeum vulgare* L.), triticale (× *Triticosecale* Wittm. ex A. Camus) and pearl millet (*Pennisetum glaucum*) and their performance were compared to the traditional forage crops such as alfalfa (*Medicago sativa* L., 1753) and forage corn (*Zea mays*). The finding of this research revealed the high potential of alternative forage crops, especially blue panicum that recorded the highest fresh yield exceeding 100 t·ha^-1^ (under experimental conditions) under salinity conditions bypassing the alfalfa and forage corn forage yield by almost two and four times, respectively.

Blue panicgrass or blue panicum was introduced in Morocco and more precisely in the Foum Eloued area, region of Laayoune, since 2016, and now appears to be alternative forage crop under irrigated salt-affected conditions of the southern part of the country (Hirich et al., 2021). The grass is a native of India, Australia, Afghanistan, and Pakistan. It is a perennial and tall growing bunch of grass (> 2 m height under optimal growing conditions). The grass is a vigorous, coarse, branching plant and produces many leaves of 1 to 2 cm wide, 30 to 50 cm long and normally blue-green in color. The plant develops a strong rhizomatic root system, especially after several cuttings, allowing it to be more resistant to water deficit (Maki, 1966).

Several studies reported that blue panicgrass is a highly resistant crop to salinity and can produce a satisfactory yield under high salinity conditions. For instance, Ashraf (2003) reported that the shoot dry weight of 3 populations (normal, waterlogging tolerant and salt-tolerant) of blue panicgrass, irrigated with saline water with a NaCl concentration of 200 mol.m^-3^ exhibited a reduction of about 59, 36, and 61%, respectively compared to control. However, the salt-tolerant population recorded the highest biomass yield under salinity conditions, about 51% more than the other two tested populations. Another study by Farrag et al. (2021) showed that blue panicgrass yield was only reduced by 40% under high salinity with a salt concentration equal to 15000 mg.l^-1^ compared to control irrigated with freshwater. Adjacent to the experimental site of the present work in the Souss Massa region (central Morocco), Oumasst et al. (2021) irrigated blue panicgrass in a pot experiment with four salinity levels (1, 3, 6, and 10 dS.m^-1^). Their obtained results revealed no significant difference between the tested salinity levels in terms of biomass yield which indicate that the salinity tolerance threshold of blue panicgrass is probably beyond 10 dS.m^-1^. In a recent study conducted under similar pedo-climatic conditions as the present experiment (Foum Eloued), Bouras et al. (2022) reported that the annual fresh biomass yield was reduced by 18% and 25% under irrigation water salinity of 12 and 17 dS·m^−1^, respectively, compared to the control.

A few studies have determined the optimal cutting time intervals of blue panicgrass when are cultivated in saline conditions. With the current expansion and upscaling process of the crop in several salt-affected areas of Morocco and elsewhere in the similar desertic conditions, it is necessary to provide farmers with scientific feedback to the question at which time interval and frequency is optimal for cutting blue panicgrass with highest forage biomass yield and nutrient content. Therefore, this study aims to determine the blue panicgrass growth, productivity, and forage quality as subjected to multiple cutting time intervals in the desert climate conditions.

## 2 Materials and methods

### 2.1 Experimental site

The field trial was carried out at the experimental farm in the Foum Eloued region, near Laayoune, Morocco (latitude = 28.1862; longitude = -13.3425; altitude = 14 m asl). The chemical and physical characteristics are presented in **Table 1**. The soils have a sandy loam texture and highly saline (EC_1:5 = _5.54 dS/m).

**Table 1:** Initial soil physical and chemical properties in the experimental site.

Sand	Silt	Clay	pH	EC_1:5_	N	P	K	Na	Ca	Mg	Zn	Mn	Cu	B
%				dS/m	%	g/Kg					mg/Kg			
66	22	12	7.91	5.54	0.08	0.84	1.5	3.43	4.69	8.9	27.85	129.63	8.64	25.74

**Figure 1:**
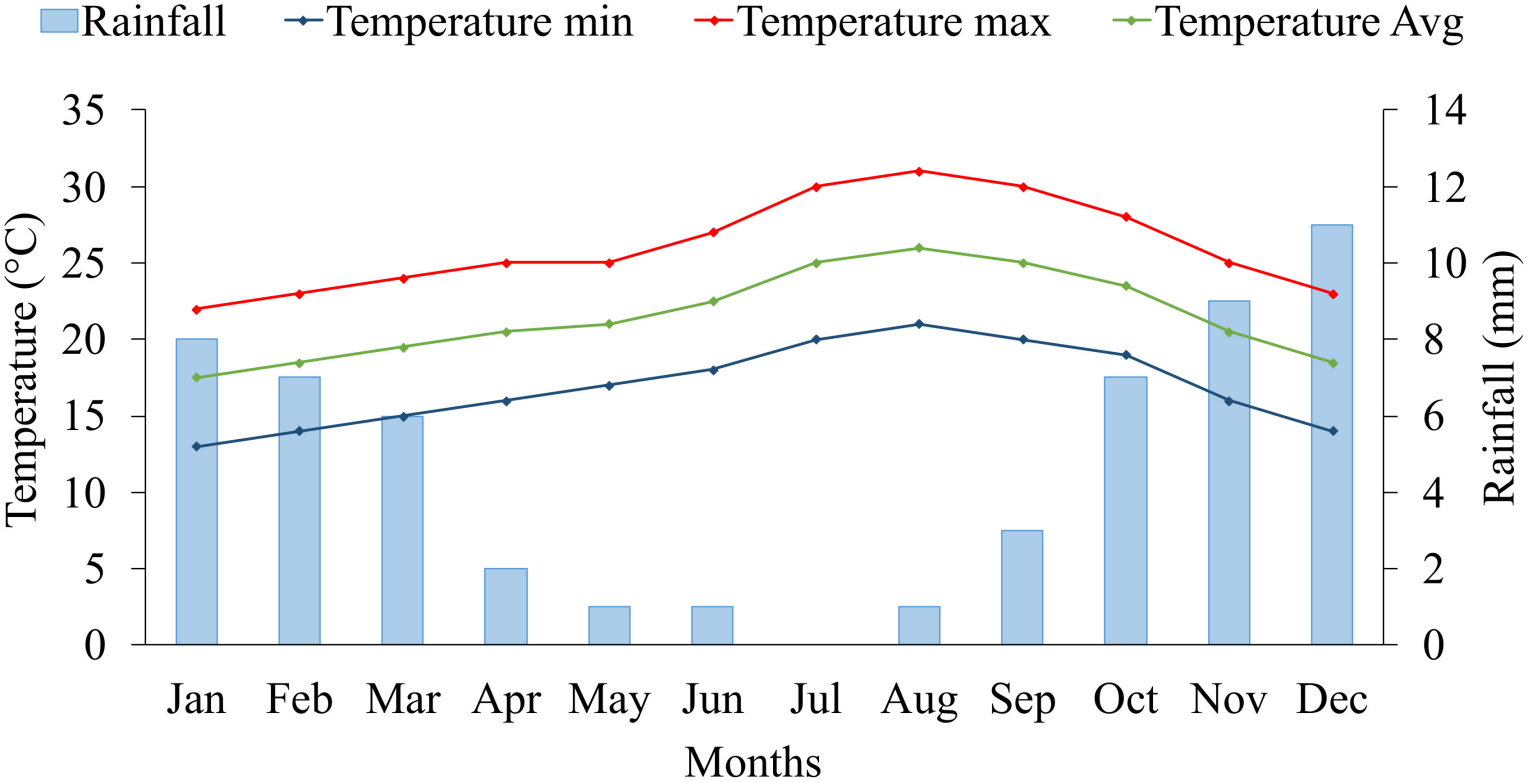
Monthly average rainfall (mm), maximum, minimum, and mean temperature (°C) at the experimental site (2017-2021) in Foum Eloued, Laayoune, Morocco.

The five years average (2017-2021) of climate data on the experimental site is presented in **Figure 1**. The experimental site is characterized by a very dry desert climate, with an average annual rainfall of 56 mm. Regarding temperature records, we defined two growing seasons for blue panicgrass: the cold season (winter), from November to March, with a maximal and minimal average temperature below 25 and 15°C, respectively, and the warm season (summer) from April to October, with a daily average temperature above 20°C. Furthermore, the cold season is also characterized by a daytime shorter than 12 hours.

### 2.2 Design of the experiment, treatments, and agricultural practices

The experiment was conducted on a Latin square design with five technical replications. Treatments included five cutting time intervals of 20, 40, 60, 80, and 100 days, as well as two contrasting growth seasons (winter and summer) were investigated. Plant materials consist of a public variety of blue panicgrass (*Panicum antidotale* Retz.) introduced from Kuwait. The crop was first sown in March 2018, and the monitoring was performed on the third-year cycle of the grass between July 2020 and September 2021. To homogenize the field grass, a first harvest was made 20 days before the start of the experiment. The plots consisted of seven rows of blue panicgrass, with an individual plot area of 12 m². The crop was irrigated using a drip irrigation system with 50 cm row-to-row spacing and a 25 cm distance between the integrated drippers (2 L/h flow). The crop received 440 mm of irrigation water during the study period. Irrigation water characteristics are presented in **Table 2**, where the applied groundwater was highly saline (16.3 dS/m), with an excessive amount of chloride and sodium.

**Table 2:** Chemical properties of irrigation water applied.

ECiw	pH	Na	K	Ca	Mg	Cl	SO4	NO3	HCO3
dS/m		mg/Kg							
16.3	7.41	2202.04	101.65	804.28	274.1	4128.21	2772	55.94	326.96

### 2.3 Measurements and plant analysis

#### 2.3.1 Agro-morphological traits

Five individual plants per plot were sampled randomly from each corresponding plot to assess the agro-morphological traits of blue panicgrass each 20 days during the entire cropping season. The whole plants were evaluated for plant height measurement, tillers and panicles count, and fresh and dry biomass weight. Additionally, five individual stems were selected randomly from the harvested plants to determine the number of leaves, number of branches, panicle length, and the individual stem and panicle fresh and dry weight. Fresh and dry biomass was determined by harvesting and weighing the whole plot. The dry matter was determined for stems, panicles, and entire plant samples by oven drying at 60°C until reaching a constant dry weight. At the end of the experiment, root samples for each cutting time interval treatment were collected to determine root length, number of rhizomes, and dry biomass.

#### 2.3.2 Mineral nutrients accumulation and partitioning

The dried shoot and root samples were finely ground and sieved through a 2 mm pore sieve. Shoot samples were collected respecting the harvest calendar for each corresponding cutting treatment interval after harvest, whereas root samples were collected at the end of the experiment. Five homogenized samples were selected to represent the cutting time interval in each season. Total plant nitrogen content was determined following the Kjeldahl (AACC International, 2000) method. Samples were further prepared by adding 8 ml of nitric acid (HNO_3_) to the grounded material (0.5 g), it was placed in a digestion tube and left overnight. The mixture was then heated at 90°C for 60 minutes and 3-4 ml of 30% hydrogen peroxide (H_2_O_2_) was added. The digestion process was stopped once the solution became colorless, and after cooling, the digest was filtered and diluted with hydrochloric acid (HCl). The elemental determination of minerals (P, K, Ca, Na, and Cl) in extracted samples was carried out by using the Inductively Coupled Plasma-Optical Emission Spectrometry method (Pequerul et al., 1993).

#### 2.3.3 Forage quality

The same five composite samples used for mineral nutrients composition were subjected to several analyses to assess the forage quality of blue panicgrass. First, ash content was determined by incinerating the samples in a muffle furnace at 550°C for 4 h. Subsequently, forage fiber components were determined using FIWE Fiber Analyzer (VELP Scientifica, FIWE 6, Italy) following the Weende procedure for crude fiber content, while Van Soest method was used for neutral detergent fiber (NDF), acid detergent fiber (ADF), and acid detergent lignin (ADL) (Van Soest et al., 1991). In addition, fats were determined by placing 5 g of the grounded forage material in a filter paper cartridge and defatted using the Soxhlet apparatus with Hexane (1:10 w/v) as solvent. The crude protein was derived from 6.25 times the nitrogen concentration obtained from the Kjeldahl procedure. Overall crude protein yield per hectare was estimated by multiplying dry biomass production by the crude protein content.

### 2.4 Statistical analysis

Statistical analysis was performed in R (R Core Team, 2022). Analysis of variance (ANOVA) was conducted to compare the means of measured and analyzed parameters for each treatment. Further, significantly different means were subjected to multiple pairwise comparisons using the Fisher Least Significant Difference test (LSD) at *p* < 0.05. Pearson’s correlation matrix was performed to investigate the strength of the linear relationship between variables, while those parameters were visualized using the “corrplot” R package (Wei and Simko, 2021). To assess the diversity and relationships of cutting time intervals with their corresponding traits, the principal component analysis (PCA) was used to investigate the correlation among the traits and identify clusters, thereby assisting in analyzing the patterns of variation among the applied treatments.

## 3 Results

### 3.1 Influence of season on growth and development stages

**Figure 2:**
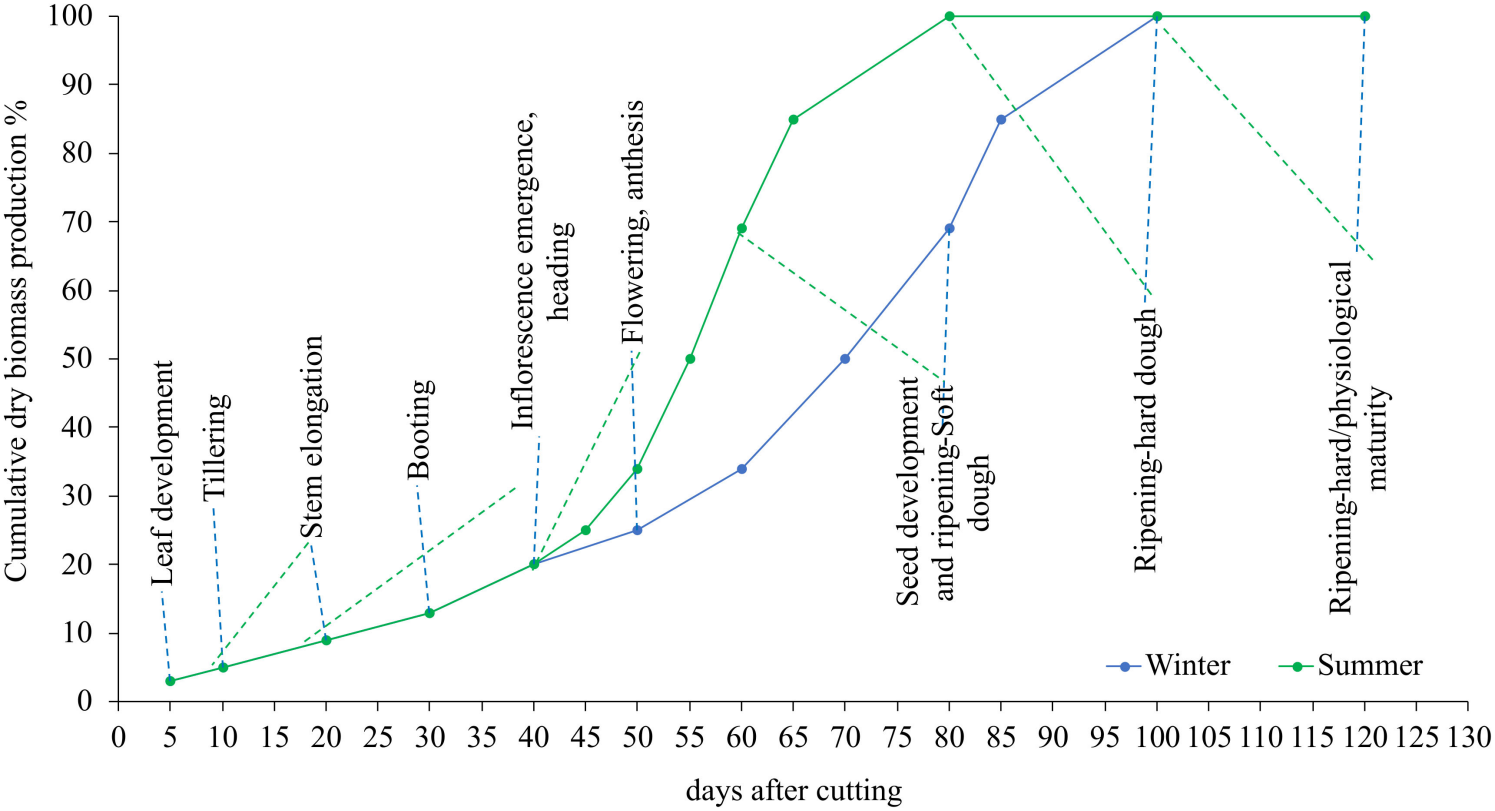
Influence of season and cutting intervals on growth and development stages and estimated cumulative dry biomass production (%) in blue panicgrass.

**Figure 2** shows the variation of the cumulative dry matter evolution during the summer and winter growing cycles. The results show that plants developed swiftly throughout the summer season due to the fast accumulation of fast-degree days. In the summer, for example, blue panicgrass began flowering 20 days after being clipped. Plants reach this stage after 40 days in the winter. In general, we observed a 20-day disparity in growth and development phases between summer and winter.

### 3.2 Agro-morphological parameters

The variation of different agro-morphological traits of blue panicgrass as affected by cutting time intervals and crop season is presented in **Table 3**. Our study found a significant difference (*p* < 0.001) between tested cutting time intervals in both growing seasons conditions for the evaluated parameters. In general, most of the traits increased in their equivalent values with the increase of cutting time intervals following a linear trend. On the other hand, the crop development rate was higher during the summer season, while it produced less biomass during the winter season due to its adaptation to hot climate. Conversely, blue panicgrass developed more tillers during the winter season compared to the summer especially under 80- and 100-day intervals.

**Table 3:** Effect of cutting time intervals and crop season on agro-morphological parameters of blue panicgrass.

Season	Summer						Winter				
Cutting time intervals	20 days	40 days	60 days		80 days	100 days	20 days	40 days	60 days	80 days	100 days
Whole plant traits											
Plant height (cm)	30.74 ± 0.51 e	67.28 ± 1.01 d	100.10 ± 1.02 c		106.09 ± 1.18 b	117.41 ± 1.27 a	23.37 ± 0.58 e	47.3 ± 1.09 d	58.75 ± 1.95 c	92.98 ± 1.6 b	103.16 ± 1.44 a
Number of tillers	23.92 ± 0.56 c	34.71 ± 1.13 b	38.91 ± 1.98 a		40.87 ± 1.61 a	39.76 ± 2 a	24.82 ± 0.92 c	32.26 ± 1.52 b	33.14 ± 2.27 b	49.66 ± 3.41 a	44.34 ± 2.98 a
Number of panicles	5.7 ± 0.43 e	20.36 ± 1.12 d	36.04 ± 2.23 c		47.03 ± 2.6 b	60.94 ± 3.8 a	6.58 ± 0.61 e	13.71 ± 1.11 d	26.88 ± 3.44 c	46.6 ± 4.24 b	55.98 ± 3.35 a
Plant fresh weight (g/plant)	105 ± 10.7 d	286.8 ± 29.6 c	465.5 ± 42.1 b		484.5 ± 48.2 b	675.5 ± 59.5 a	87.41 ± 9.63 d	151.4 ± 12.1 c	252.7 ± 40 b	432.7 ± 66.4 a	454.5 ± 27.5 a
Plant dry weight (g/plant)	28.25 ± 3.15 e	82.86 ± 8.76 d	151.5 ± 13.7 c		186.3 ± 17.9 b	298.1 ± 28.4 a	22.72 ± 2.5 e	44.5 ± 4.35 d	79.9 ± 13.7 c	158.1 ± 21.8 b	187.76 ± 8.01 a
Plant dry matter (%)	26.41 ± 0.49 e	28.8 ± 0.39 d	32.82 ± 0.53 c		39.47 ± 1.3 b	44 ± 0.84 a	26.8 ± 0.99 c	29.11 ± 1.3 bc	31.21 ± 0.92 b	37.67 ± 2.46 a	42.23 ± 2.24 a
Panicle traits											
Panicle length (cm)	5.23 ± 0.19 d	13.62 ± 0.28 c	18.55 ± 0.24 b		18.33 ± 0.29 b	19.6 ± 0.29 a	5.51 ± 0.2 d	9.83 ± 0.31 c	10.86 ± 0.5 b	17.8 ± 0.31 a	18.1 ± 0.27 a
Panicle fresh weight (g)	0.17 ± 0.01 d	0.57 ± 0.02 c	1.1 ± 0.03 a		0.76 ± 0.03 b	0.79 ± 0.04 b	0.12 ± 0.01 e	0.43 ± 0.03 d	0.64 ± 0.04 c	1.2 ± 0.04 a	0.8 ± 0.04 b
Panicle dry weight (g)	0.06 ± 0.01 d	0.23 ± 0.01 c	0.39 ± 0.01 a		0.39 ± 0.02 a	0.31 ± 0.04 b	0.06 ± 0.01 e	0.18 ± 0.01 d	0.26 ± 0.02 c	0.49 ± 0.02 a	0.33 ± 0.02 b
Main stem traits											
Number of branches/stem	0 ± 0 e	0.15 ± 0.03 d	1.91 ± 0.11 c		3.36 ± 0.14 b	3.84 ± 0.17 a	0 ± 0 c	0 ± 0 c	1.67 ± 0.14 b	2.86 ± 0.16 a	3.1 ± 0.17 a
Number of leaves/stem	7.89 ± 0.1 e	11.39 ± 0.17 d	19.31 ± 0.61 c		27.37 ± 0.89 b	30.68 ± 1.15 a	7.02 ± 0.12 e	10.5 ± 0.22 d	15.1 ± 0.69 c	20.89 ± 0.72 b	26 ± 0.88 a
Stem fresh weight (g/stem)	2.68 ± 0.06 d	4.82 ± 0.13 c	7.04 ± 0.19 b		7.19 ± 0.21 b	8.37 ± 0.25 a	1.59 ± 0.06 e	3.56 ± 0.13 d	4.28 ± 0.16 c	6.29 ± 0.22 b	7.22 ± 0.26 a
Stem dry weight (g/stem)	0.87 ± 0.03 c	1.46 ± 0.04 b	2.4 ± 0.08 a		2.42 ± 0.07 a	2.37 ± 0.09 a	0.44 ± 0.02 e	1.11 ± 0.04 d	1.29 ± 0.05 c	1.84 ± 0.06 b	2.77 ± 0.13 a

Values represent mean ± standard error. Different letters indicate a significant difference between cutting time intervals on each season at *p* < 0.05 level of significance using the Fisher LSD test.

### 3.3 Final root development

**Figure 3:**
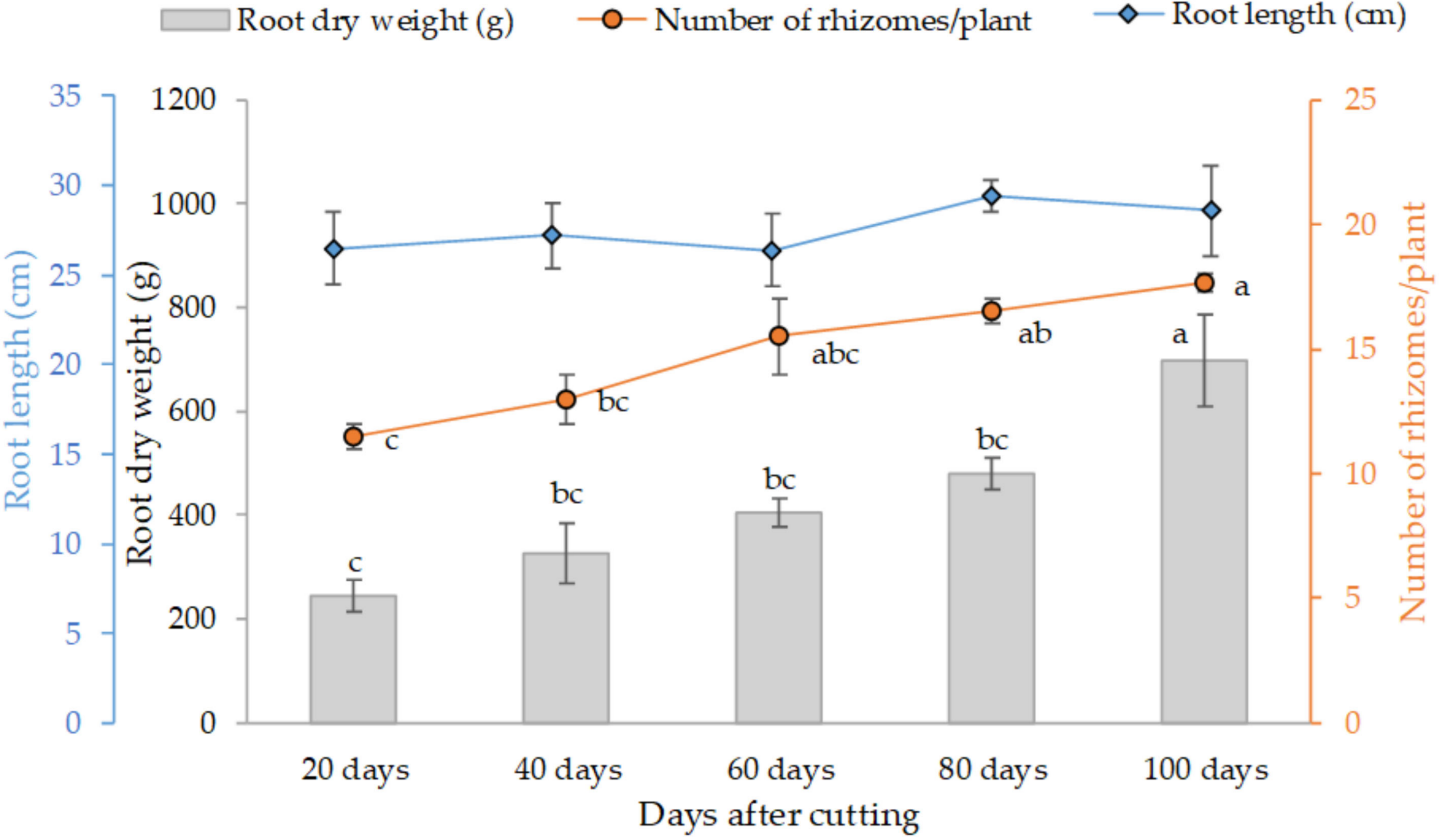
Effect of cutting time intervals on variation of final root weight, number of rhyzomes per plant and average root length. Values represent mean ± standard error. Different letters indicate a significant difference between cutting time intervals on each season at p < 0.05 level of significance using the Fisher LSD test.

**Figure 3** presents the variation of the final root weight, number of rhizomes per plant and average root length as affected by different tested cutting time intervals. The trend clearly shows that individual root weight and their proliferation increased with cutting time intervals, at subtle rates in 20 (245 g) to 80 days (480 g), and distinctly greater values at the 100-day interval with an average of 700 g of dry weight per individual plant. Statistical analysis also indicates a similar trend for the development of rhizomes. However, root length was not affected by the cutting time intervals, and the average length was about 28 cm for all treatments.

### 3.4 Productivity parameters

#### 3.4.1 Average biomass per cut

**Figure 4:**
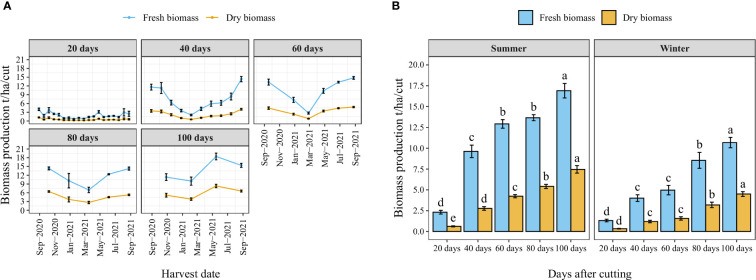
Year-round evolution of biomass production of blue panicgrass for different cutting time intervals **(A)**, and variation of average fresh and dry biomass per cut according to different cutting time intervals and seasons **(B)**. Values represent mean ± standard error. Different letters indicate a significant difference between cutting time intervals on each season at p < 0.05 level of significance using the Fisher LSD test.

**Figure 4A** illustrates the year-round record of fresh and dry biomass production for each cutting time interval as well as the number of cuts per year. The trend indicates a decrease in biomass production during the winter season (from November to March). Nevertheless, the high temperature of the summer significantly improved biomass production. Similarly, the average fresh and dry biomass per cut increased with the increased cutting time interval following a linear trend where the highest biomass weight per cut was obtained when a time interval of 100 days was kept between two cuts (**Figure 4B**). Regarding the effect of season, biomass production was significantly reduced during the winter season, where average fresh biomass production per cut was reduced in the winter season compared to the summer season by 43, 58, 62, 38, and 37% under cutting time intervals of 20, 40, 60, 80, and 100 days, respectively.

#### 3.4.2 Annual forage yield

**Figure 5:**
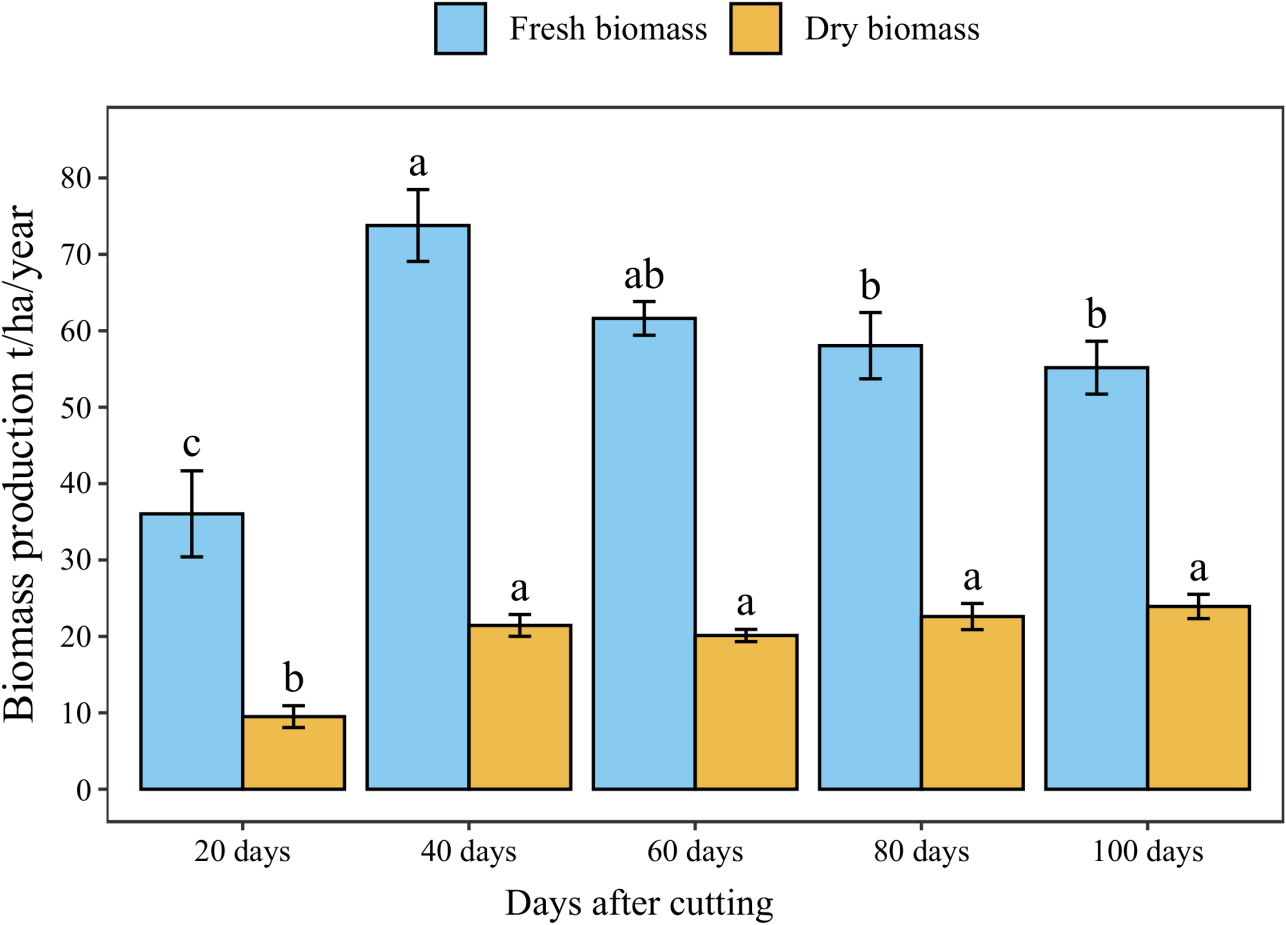
Annual forage yield of blue panicgrass at different cutting time intervals. Values represent mean ± standard error. Different letters indicate a significant difference between cutting time intervals on each season at *p* < 0.05 level of significance using the Fisher LSD test.

The annual accumulated fresh and dry forage yield under different cutting time intervals is presented in **Figure 5**. The statistical analysis showed a significant difference (*p* < 0.05) between different intervals revealing four distinct groups in terms of fresh biomass. The highest cumulative annual forage yield (74 t/ha) was recorded under 40-day cutting interval, followed by the 60-day interval (62 t/ha). Whereas the lowest forage yield was obtained under a 20-day cutting time interval (36 t/ha) with a reduced rate of 56% compared to 40-day interval. On the other hand, harvesting blue panicum every 40 to 100 days was not statistically different in dry biomass, even though it ranged from 20 to 24 t/ha. While cutting the plant every 20 days resulted in a significant reduction by 61% of aboveground dry biomass accumulation compared to the 40-day interval.

### 3.5 Mineral nutrients accumulation and partitioning

**Figure 6:**
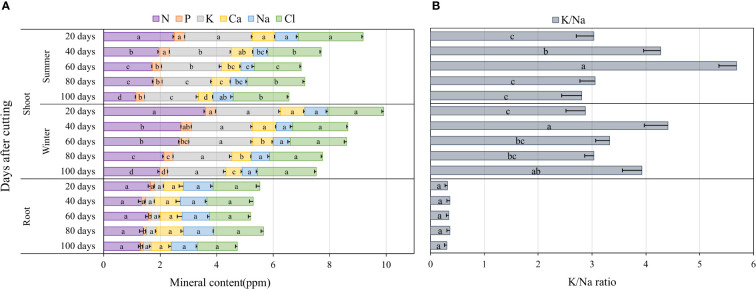
Nutrient accumulation in shoots and roots as affected by cutting time intervals and season **(A)**, and variation of K+/Na+ in blue panicgrass shoots and roots as a response to cutting times intervals and season **(B)**. Values represent mean ± standard error. Different letters indicate a significant difference between cutting time intervals on each season at p < 0.05 level of significance using the Fisher LSD test.

The accumulation of major nutrients such as nitrogen (N), phosphorus (P), potassium (K), calcium (Ca), sodium (Na) and chloride (Cl) in blue panicgrass shoots and roots as affected by both cutting time intervals and season are presented in **Figure 6A**. Nutrient content on the shoot biomass was significantly affected by cutting time intervals during the summer season (*p* < 0.01), while during the winter, cutting time intervals were significantly different (*p* < 0.05) only for N, P, and Ca. In fact, an increase in the cutting time interval resulted in a reduction of the nutrients content of the investigated minerals in shoot biomass. The root mineral composition, with the exemption of P, was not affected by the cutting interval. Regarding seasonal effect, plants accumulated greater amount of N in their shoot during winter compared to summer. In general, N, P, and K content in shoots was much higher in leaves compared to roots, whereas Na content was higher in roots (1 ppm) compared to shoots (0.63 ppm) on average of all investigated treatments.

To understand better the partitioning of Na and K which two important elements are involved in plant response to salinity where Na is considered a toxic element and K a compatible osmolyte for plant metabolism, we determined the K^+^/Na^+^ ratio as a key indicator for plant tolerance to salinity. Our data presented in **Figure 6B** indicate clearly that the average K^+^/Na^+^ ratio in the shoots is about 6 to 15 times more than the ratio in roots. Furthermore, the highest ratio in the shoots was observed under 40- and 60-day cutting time intervals during the winter and the summer season, respectively.

### 3.6 Forage quality

Analysis of variance revealed a significant difference in forage nutritive value determinants of blue panicgrass across cutting time intervals and seasons (**Table 4**). The forage composition of ash, crude protein, and fats decreased with the increase of cutting interval. However, crude fibers, neutral detergent fiber (NDF), acid detergent fiber (ADF), and acid detergent lignin (ADL) significantly increased along increasing cutting interval. The highest value for crude protein was recorded under 20 days intervals with 15.6 and 22.5% of dry matter during the summer and the winter seasons, respectively. Furthermore, the 100-days cutting interval recorded the highest value in terms of crude fibers with 31.8 and 31.3% of dry matter respectively for the summer and winter seasons.

**Table 4:** Effect of different cutting time intervals and crop season on forage quality parameteres of blue panicgrass.

Season	Summer					Winter				
Cutting time intervals	20 days	40 days	60 days	80 days	100 days	20 days	40 days	60 days	80 days	100 days
**Ash (%)**	12.65 ± 0.19 a	11.5 ± 0.2 b	10.46 ± 0.16 c	10.32 ± 0.14 cd	9.84 ± 0.21 d	13.15 ± 0.34 a	12.43 ± 0.16 b	12.25 ± 0.25 b	10.96 ± 0.19 c	10.9 ± 0.19 c
**Crude protein (%)**	15.6 ± 0.22 a	12.3 ± 0.27 b	10.89 ± 0.18 c	11.07 ± 0.29 c	7.18 ± 0.21 d	22.53 ± 0.22 a	17.21 ± 0.14 b	16.84 ± 0.2 b	13.29 ± 0.23 c	12.38 ± 0.3 d
**Crude fiber (%)**	24.24 ± 0.63 c	28.18 ± 0.36 b	31.26 ± 0.39 a	31.67 ± 0.98 a	31.78 ± 0.48 a	21.65 ± 0.45 d	23.65 ± 0.37 cd	25.04 ± 0.4 c	27.96 ± 0.89 b	31.32 ± 1.05 a
**NDF (%)**	52.91 ± 0.88 d	55.56 ± 0.68 c	58.27 ± 0.32 b	58.95 ± 0.64 b	62.02 ± 0.8 a	48.77 ± 0.73 c	52.51 ± 0.24 b	53.84 ± 0.57 b	59.47 ± 0.56 a	60.57 ± 0.5 a
**ADF (%)**	27.96 ± 0.67 d	29.94 ± 0.48 c	31.7 ± 0.2 b	32.06 ± 0.29 b	34.05 ± 0.12 a	24.61 ± 0.31 c	26.32 ± 0.24 b	26.84 ± 0.22 b	31.32 ± 0.49 a	32.4 ± 0.91 a
**ADL (%)**	2.98 ± 0.14 d	3.29 ± 0.28 d	4.54 ± 0.43 c	5.94 ± 0.6 b	8.96 ± 0.49 a	2.73 ± 0.18 b	3.75 ± 0.51 b	5.14 ± 0.26 a	5.58 ± 0.47 a	5.81 ± 0.45 a
**Fat (%)**	2.82 ± 0.12 a	3.02 ± 0.07 a	2.94 ± 0.05 a	2.28 ± 0.19 b	2.13 ± 0.15 b	3.31 ± 0.15 a	2.84 ± 0.13 b	2.83 ± 0.15 b	2.61 ± 0.13 b	2.15 ± 0.11 c

Values represent mean ± standard error. Different letters indicate a significant difference between cutting time intervals on each season at *p* < 0.05 level of significance using the Fisher LSD test.

### 3.7 Crude protein yield

**Figure 7:**
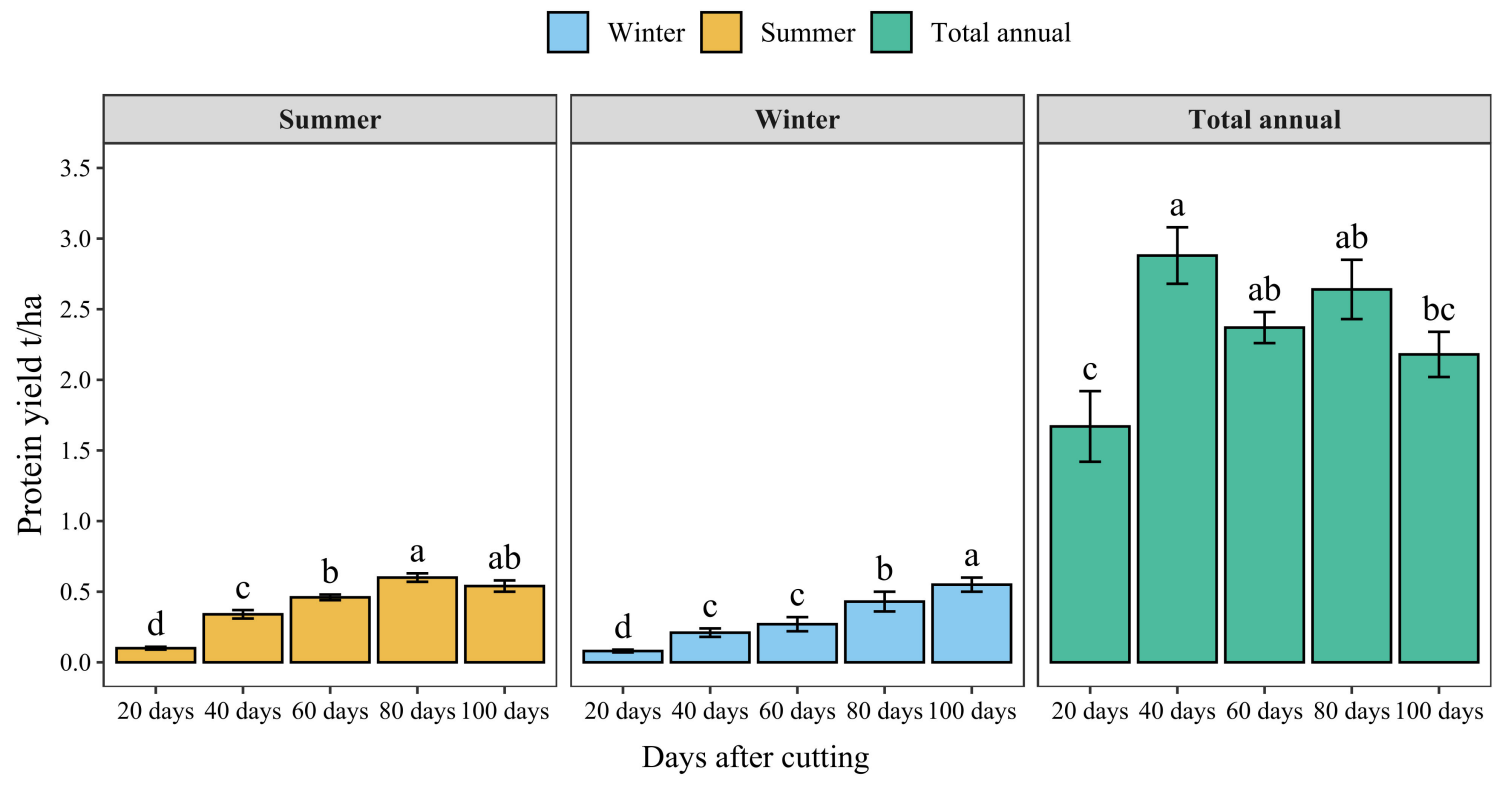
Crude protein yield of blue panicgrass as affected by cutting time intervals and seasons, and its cumulative annual yield. Values represent mean ± standard error. Different letters indicate a significant difference between cutting time intervals on each season at *p* < 0.05 level of significance using the Fisher LSD test.

**Figure 7** presents the protein yield of blue panicgrass by cut during both summer and winter seasons and its annual cumulative production. Results indicate a significant increase in protein yield with increased cutting time intervals. However, the highest protein yield by cut was recorded for 80-day cutting intervals during the summer season (0.6 t/ha/cut of crude protein). Regarding annual protein production, cutting blue panicgrass every 40 days had the highest annual production with 2.9 t/ha/year, while 20 days interval exhibited the lowest value (1.7 t/ha).

### 3.8 Correlation matrix

**Figure 8:**
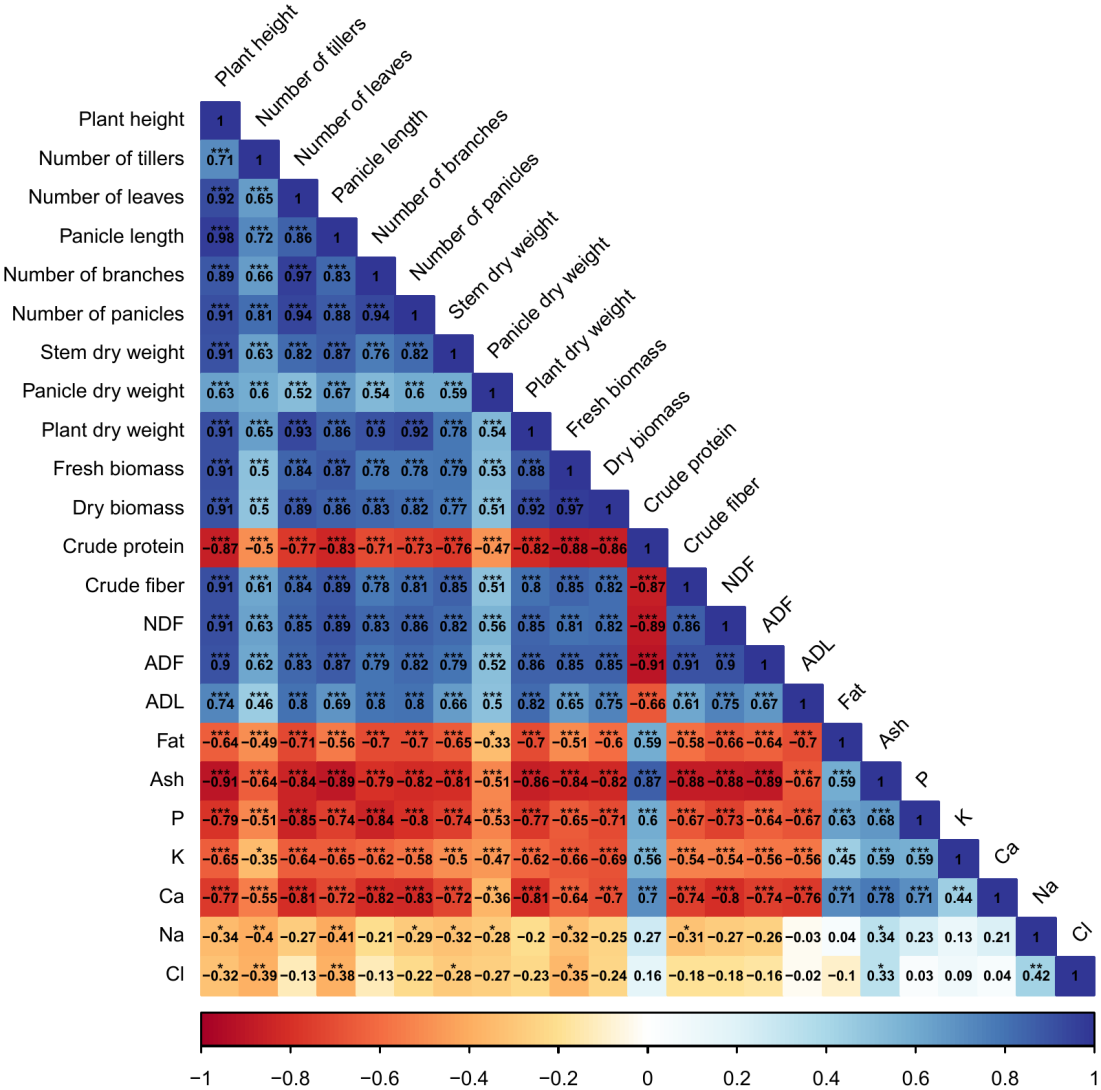
Pearson’s correlation matrix for the investigated parameters. Values in the matrix represent Pearson’s correlation coefficient. *, **, *** indicate the significance of the correlation coefficient at *p* < 0.05, 0.01, and 0.001, respectively.

Correlations among agro-morphological, productivity measurements, minerals content, and forage nutritive values are presented in **Figure 8**. Results indicate a significant (*p* < 0.001) and strong positive correlation between agro-morphological traits, fresh and dry biomass production, crude fiber, NDF, ADF, and ADL. However, crude protein content had a highly significant (*p* < 0.001) and strong negative correlation with dry biomass production (-88%). Moreover, a negative relationship was also revealed between mineral content and agro-morphological and productivity traits.

### 3.9 Principal components analysis

**Figure 9:**
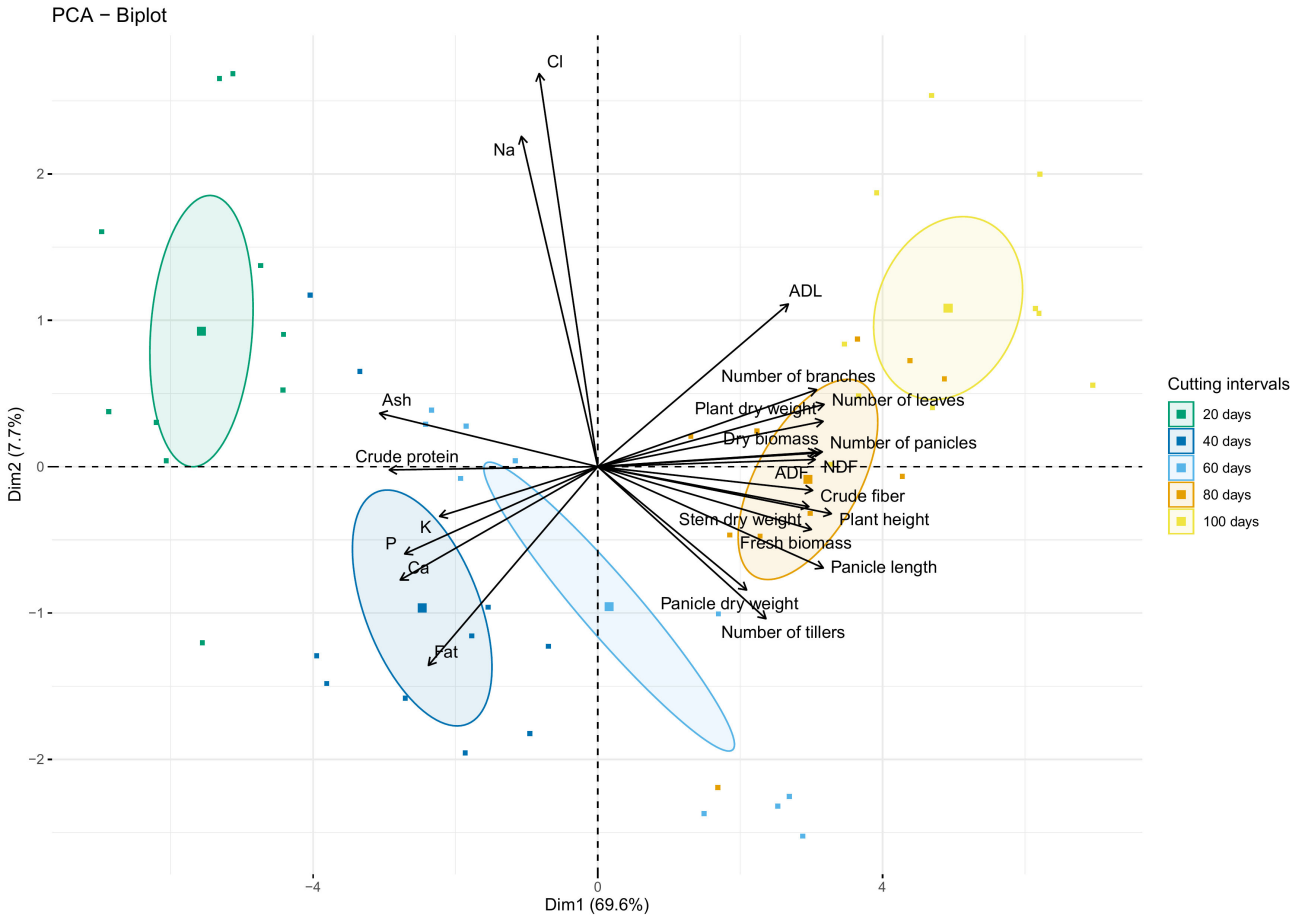
PCA-biplot of all investigated parameters. Arrows and square marks indicate variables and individuals’ projection, respectively. Circles indicate the centroid of the corresponding treatment clusters.

**Figure 9** shows the biplot of agro-morphological measurements and nutrient content as affected by cutting interval analyzed using the principal component analysis (PCA). Results indicate that the first two dimensions explain 77.3% of the data variability. The first dimension (Dim 1) exhibited about 70% of this total variability and was explained principally by crude protein, ash, crude fiber, NDF, ADF, and all agro-morphological traits except the number of tillers, which contribute to the formation of the second dimension (Dim 2), with ADL, Fat, Na and Cl.

PCA biplot analysis revealed that crude protein and ash contents were strongly correlated with the cluster of individuals subjected to 20- and 40-days cutting intervals. However, these parameters present a strong dissimilarity with the cluster of 80- and 100-days cutting intervals, characterized mainly by high fiber content and almost agro-morphological and productivity parameters. Moreover, the cluster group for 60 days cutting interval is mainly associated with traits of the second dimension (Dim 2), while individuals negatively correlated with the amount of Na and Cl.

## 4 Discussion

### 4.1 Biomass accumulation and crop growth

The finding of this study indicated that increasing cutting time interval increased the biomass accumulation per cut as well as most of the other investigated morphological traits. There are few studies that investigated the effect of cutting (clipping) time intervals or frequency on blue panicgrass, and our findings are in line with several studies. For instance, Sarwar et al. (2006) reported that increasing cutting intervals resulted in increased productivity and dry matter percentage where dry biomass yield increased by 2.78 and 4.12-times under 60- and 120-day intervals compared to 30-days. More or less the same increase percentage was obtained in our study where dry biomass yield increased by 2.75 and 3.92-times under 60- and 100-day intervals compared to 30-days (taking into account the average values of dry matter yield of 20- and 40-day intervals). In a recent study conducted by Habib et al. (2019), it was reported that the significant effect of cutting time interval on biomass accumulation was only obvious under 80-days interval while no significant difference was observed under 20-, 40-, and 60 days. Conversely, our study proved that there had a very good linear relationship between cutting interval and biomass accumulation. Similarly to our finding, Mutz and Drawe (1983) found that the highest dry matter yield was achieved when clipping blue panicgrass every 8 weeks (2 months) with an increase of 25% compared to 4 weeks cutting time intervals. While the lowest dry matter yield was recorded when cutting the plant once a year. Mirza et al. (2002) reported a similar trend where the annual dry matter yield of five types of grass including blue panicgrass was maximal at the flowering stage (which is 40-days cutting time interval in our case) while the lowest yield was recorded during the maturity stage.

For many types of grass, tiller development indicates the number of shoots produced and, thus, biomass production. Since a tiller can be defined as an axillary shoot growing from the axis of the leaf, then the potential production of tillering depends on the number of leaves produced (Bashir, 1982). It is widely recognized that the removal of apical growth dominance through cutting induces tiller production, but still tillers will not be developed unless food reserves are enough. Our finding agreed with Bashir (1982) who reported that increasing the cutting time intervals (15-, 30 and 45-days) resulted in more production of forage mainly through the increased stem height and the number of tillers. In another study testing the responses of switchgrass (*Panicum virgatum* L.), which belongs to the same genus of blue panicgrass, to three cutting time intervals, Madakadze et al. (1999) reported that biomass and tiller production increased as a response to increased cutting date. Similarly, tiller density increased in Buffel grass (*Cenchrus ciliaris* L.) with advancing grass age (Butt and Ahmad, 1994). Contrary to our finding, Habib et al. (2019) found that the number of tillers decreased with increased cutting time intervals and this reduction may be explained by the self-shading effect of mature plants. Butt and Ahmad (1994) also found that increased harvest frequency (one, two, three, or four harvests per season) reduced the total seasonal biomass yield of switchgrass; however, the final root weight increased. They suggested that switchgrass remobilized and translocated storage compounds from leaves and stems to the roots which were partially inducing yield loss and increased root biomass accumulation. In our case, this explanation can be valid for the cutting time intervals starting from 40- to 100-days where partial biomass yield loss under 80- and 100-day intervals can be also attributed to plant drying with advanced age and senescence occurrence. In general, for 20-day cutting time intervals, the reduced biomass production recorded can be explained by the fact that plants subjected to higher defoliation intensities have less remaining foliar area, thus less reserve accumulation and translocation compared to other cutting stages (Silveira et al., 2010).

This study revealed a 20-day difference in crop stages, maintaining the same cutting time interval between summer and winter seasons, where the plant grows rapidly during the summer season compared to winter. During the summer, when the temperature is optimal (around 28-29°C) and radiation and light interception are high, plants quickly accumulate the number of degree days needed to reach the next stage (Ahmad et al., 2017). Moreover, the photosynthetic activity during the summer increases for C4 grasses which leads to high biomass accumulation.

### 4.2 Mineral nutrients accumulation and partitioning

The finding of this study indicates that blue panicgrass subjected to salinity stress maintains a high K^+^/Na^+^ ratio in the shoots and a low ratio in the roots. Even though the irrigation water salinity in our case was mainly due to high content in Na^+^ and Cl^-^ compared to K^+^. It is well known that excessive content of Na^+^ in the plant tissues has an antagonistic effect on K assimilation and only salt-tolerant crops are able to exclude Na^+^ from the cytosol and translocate it to roots (Al-Ghumaiz et al., 2017). In addition, to maintain an adequate high K^+^/Na^+^ ratio in leaves, Na^+^ must be excluded from photosynthetically active cells and transported via the phloem from leaves to roots tissue or accumulated in the vacuoles to avoid competition with K^+^ in plant metabolism (Keisham et al., 2018). We believe that in our case, blue panicgrass is deploying this tolerance strategy to cope with salinity stress which is already supported by previous studies suggesting that this grass successfully retains Na^+^ in the root and keeps a higher K^+^/Na^+^ ratio in the shoot (Eshghizadeh et al., 2012; Shabala and Cuin, 2008; Al-Ghumaiz et al., 2017; Chen et al., 2018). Conversely, Ahmad et al. (2010) reported that the K^+^/Na^+^ ratio reduced significantly in root and shoots in all tested populations of blue panicgrass under salinity stress compared to the control (non-saline). However, the populations from highly saline habitats showed less decrease in the K^+^/Na^+^ ratio as compared to those from mild saline habitats and the K^+^/Na^+^ ratio in roots was lower compared to shoots for most of the tested population.

**Figure 10:**
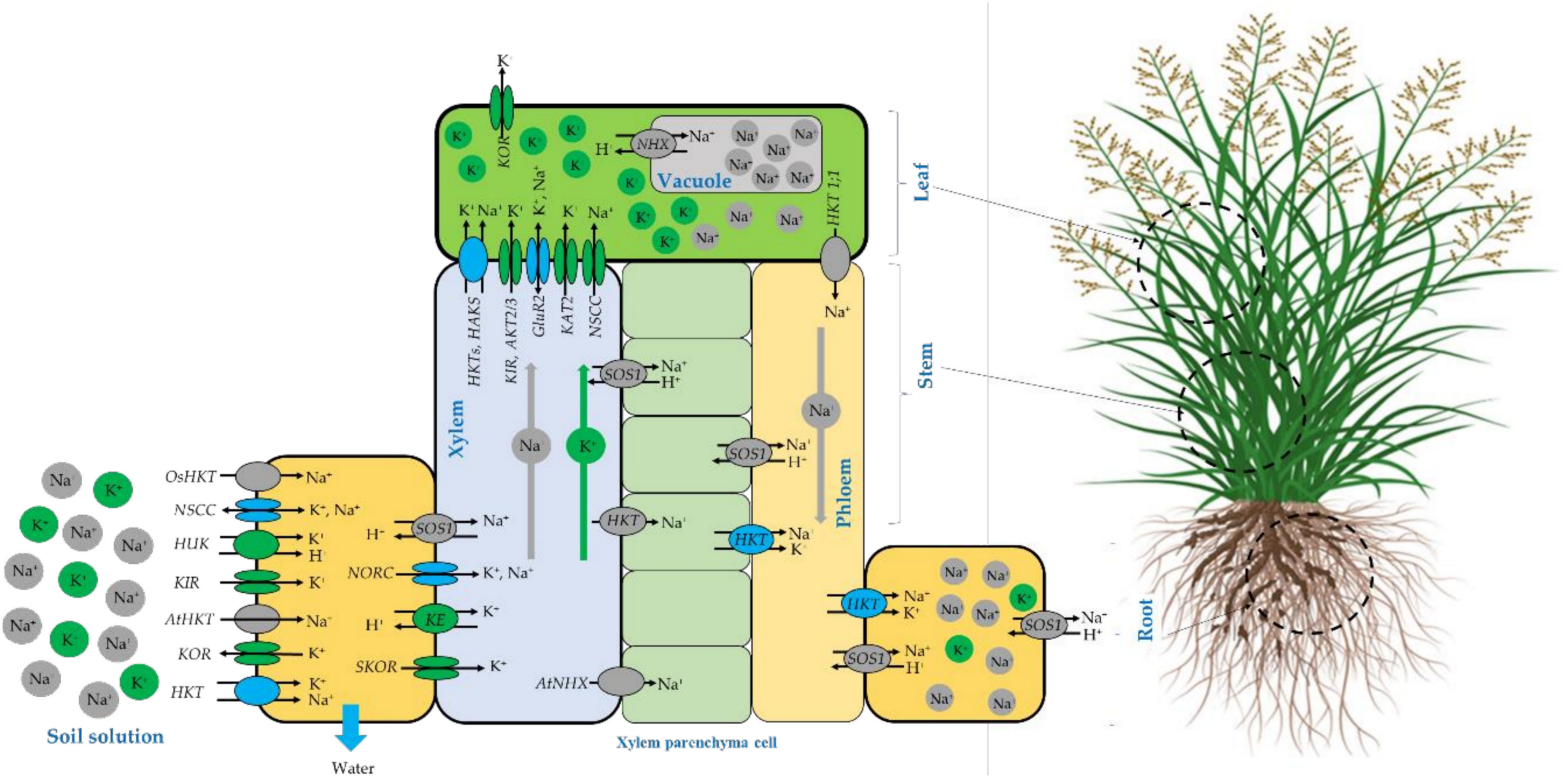
Schematic representation of N^+^ and K^+^ homeostasis and translocation in salt-tolerant plants compiled from different sources (Walker et al., 1996; Sunarpi et al., 2005; Shabala and Cuin, 2008; Nieves-Cordones et al., 2010; Núñez-Ramírez et al., 2012; Assaha et al., 2017; Chen et al., 2018; Keisham et al., 2018).

The schematic representation of Na^+^ and K^+^ homeostasis and translocation in salt-tolerant plants adapted from different sources (Sunarpi et al., 2005; Shabala and Cuin, 2008; Assaha et al., 2017; Keisham et al., 2018) are presented in **Figure 10**. It is well known that salinity affects plant growth and development by inducing both ionic and osmotic stresses and consequently driving water out of the cell to maintain water flowing inside the cell, salt-tolerant plants accumulate more compatible osmolytes such as K^+^ and Na^+^ to maintain the cytosolic water activity (Shabala and Cuin, 2008). It is also believed that the Na^+^ assimilation at the root-soil solution boundaries occurs mainly through non-selective cation channels (NSCC), as well as some high-affinity K^+^ transporters (HKTs) (Sunarpi et al., 2005; Assaha et al., 2017). However, Potassium is translocated through other channels such as voltage-dependent hyperpolarization-activated (KIR) and depolarization-activated (KOR) Shaker-type K1channels and depolarization-activated out-ward-rectifying channels (SKOR, NORK) (Shabala and Cuin, 2008). At the leaf level, Na^+^ can be transported out and in by several channels including high-affinity potassium transporters HKT (Assaha et al., 2017) and HAKS (Keisham et al., 2018), While potassium can be translocated by HKT (Walker et al., 1996), HAKS (Nieves-Cordones et al., 2010), KIR, weakly inward-rectifying channels (AKT2/3), glutamate receptors (GluRs) and hyper-polarization-activated inward-rectifying channels (KAT2) (Shabala and Cuin, 2008).

For Sodium exclusion, several mechanisms may be deployed by the plant to reduce the specific Na toxicity. At the root cellular level, Na exclusion back to the soil solution occurs through the plasma membrane salt overly sensitive (SOS1) Na^+^/H^+^ antiporter (Shi et al.,2002; Katiyar-Agarwal et al., 2006; Núñez-Ramírez et al., 2012) or just stored in the root tissues using HKT channels. Compartmentation of excessive Na^+^ in the vacuole is a salt tolerance strategy adopted by resistant species and mainly. Na^+^ is compartmentalized inside vacuoles by NHX (a Na^+^/H^+^ exchanger) as reported by (Keisham et al., 2018) and by NSCC channels (Shabala and Cuin, 2008) to avoid the antagonistic effect where the K^+^ binding sites are taken over by Na^+^ ion that consequently causes chlorophyll degradation and disruption in optimal protein functioning, this leads to photosynthetic activity alteration (Kumari et al., 2021).

In addition to the significant variation recorded for K and Na, N content in shoots significantly affected by both cutting time intervals and season. In fact, it decreased with the increase in the cutting time interval and during the summer season. However, no effect of cutting time interval was noticed for N content in the roots which in general, maintained lower compared to shoots. Our findings agree with the results obtained by (Mutz and Drawe, 1983) who reported that nitrogen content decreased by 30 and 60% when the blue panicgrass was subjected to clipping every 8 weeks and once a year respectively compared to 4 weeks. The same trends were also found for other tested grasses such as buffelgrass, bell Rhodes grass and Kleberg bluestem. (Daur, 2016) also found that nitrogen content in blue panicgrass shoots declined by 7% when harvested after flowering compared to the early stage before flowering. The same trend was found by (Mirza et al., 2002) where nitrogen content in blue panicgrass and other tested grass shoots declined with the advancement of growth stages.

It is well known that plants mobilize a higher quantity of nitrogen during vegetative stages which is necessary to produce vegetative organs such as shoots, nevertheless, N mobilization in plants decline at maturity or senescence stage as reported by several studies (Cabello et al., 2006; Diaz et al., 2008; Aziiba et al., 2019). Masclaux-Daubresse et al. (2010) explained the reduction of N mobilization in the maturity stage by the degradation of the Rubisco enzyme which is the responsible protein for N storage in plants. Our results also indicated also that at the maturity stage (100-days cutting time interval), N content in the roots during the summer season is higher than in shoots, which can be explained by nutrient mobilization from the aerial organs of the plant to the crowns and rhizomes during maturity senescence as explained by Sarath et al. (2014) and Yang and Udvardi (2018). Our study also revealed that nitrogen content in shoots was higher during the winter season compared to summer which could be explained by the negative impact of temperature on N mobilization occurring during summer. This result agreed with that obtained by Xu and Zhou (2005) who reported that high warming decreased nitrogen concentration in the plant shoots. A possible explanation suggested by Pérez et al. (2005), is that high temperatures tend to decrease the rbcS mRNA level and Rubisco protein which resulted in decreasing the N content.

### 4.3 Forage quality

The harvesting time is a significant cultural practice that has a notable impact on feed composition. Mitchell et al. (2014) reported that the delay of biomass harvesting results on a higher level of structural carbohydrates and lignin, and lower levels of protein and ash, compared to biomass collected during anthesis. With advancing maturity, blue panicgrass accumulates more cell components in plant tissues because of stem development and a decrease on the leaf to stem ratio (Sarwar et al., 2006). In addition, the nutritional value of C4 grasses experiences a rapid decline during the warm season, when they grow more rapidly as a result of increasing temperatures and day length (Virkajärvi et al., 2012).

The result of our experiment indicates that blue panicgrass forage quality is significantly affected (*p* < 0.001) by the cutting time interval treatments. However, as mentioned in the above section, the decrease in ash and crude protein content could be explained by the decline in mineral contents. Similarly to our finding, Khan et al. (2015) reported that with the increase of physiological maturity of *Panicum antidotale*, a significant decrease in crude protein and ash at 15- and 30-days intervals after regrowth. However, crude protein and ash decreased by 15 and 19%, respectively at 30 days intervals compared to 15 days. The increase in clipping frequency had decreased the crude protein content by 20 and 47% under 60- and 120-day intervals, respectively, compared to the 30 days cut (Sarwar et al., 2006). Khan et al. (2021) reported that blue panicgrass is a high protein fodder crop, and harvesting the grass 60 days after regrowth during winter produced the highest amount of crude protein (14.3%) compared to other tested grasses, including *Cenchrus ciliaris*, *Setaria anceps*, *Panicum maximum*, *Pennisetum purpureum*, *Pennisetum orientale*, and *Atriplex lentiformis*, with crude protein percentages of 8.3%, 10.4%, 11.4%, 11.1%, 8.7%, and 6%, respectively.

Regarding fiber content, our finding indicates an increase of crude fiber, NDF, ADF, and ADL, with an increase in cutting time interval. These results align well with the previous study of Khan et al. (2015), who reported that NDF and ADF contents increased by 6% for 30 days compared to 15 days. Furthermore, Daur (2016) reported that NDF and ADF content in blue panicgrass was about 59 and 31% before flowering (35 days) and increased to 72 and 38%, respectively, after flowering (45 days). A negative relationship between ash and crude protein, with crude fiber, NDF, and ADF, was reported in several other studies. According to Arzani et al. (2004), the increase of protector and firmness tissue in plants, consisting mainly of structural carbohydrates (cellulose, hemicellulose, and lignin), causes higher amounts of fiber in forage during the late plant growth, which affects the seasonal variability of forage quality between the summer and winter.

## 5 Conclusions

This study contributed to the optimization of blue panicgrass production in arid conditions and irrigated with saline water by determining the optimal cutting time interval under which forage yield and quality are maximized also addressing the impact of the season and production variation between summer and winter. The outcomes of this work respond to the initial questions made by farmers who adopted this crop: at which time interval should we cut blue panicgrass? The results of this study recommends that the cutting of blue panicgrass should be carried out every 40 days, which is every 40 days after the first cut to maximize its forage yield and quality. Furthermore, analyzing the nutrient partitioning between roots and shoots, especially sodium and potassium allowed us to better understand the salt tolerance mechanism deployed by this grass to cope with salinity stress.

## Data availability statement

The raw data supporting the conclusions of this article will be made available by the authors, without undue reservation.

## Author contributions

Conceptualization and methodology: AH, AM, and IM. Data curation: AM, IM, and AH. Investigation: AM, IM, MB, MI, and KL. Chemical analysis: AM, IM, MI, and KL, AS. Software: AM. Supervision: AH and AN. Validation: AH. Writing -Original draft: AM and AH. Writing- Review editing: KD, DA, and EA. Resources mobilization: AH and LK. All authors contributed to the article and approved the submitted version.

## References

[B1] AACC International (2000). Approved methods of American association of cereal chemists. 10th ed (St. Paul, MN: The American Association of Cereal Chemists), 46–10.

[B2] Ahmad M. S. A. Ashraf M. Ali Q. (2010). Soil salinity as a selection pressure is a key determinant for the evolution of salt tolerance in blue panicgrass (*Panicum antidotale* retz.). Flora - Morphol. Distribution Funct. Ecol. Plants 205, 37–45. doi: 10.1016/j.flora.2008.12.002

[B3] Ahmad L. Habib Kanth R. Parvaze S. Sheraz Mahdi S. (2017). “Growing degree days to forecast crop stages,” in Experimental agrometeorology: a practical manual. Eds. Ahmad L. Habib Kanth R. Parvaze S. Sheraz Mahdi S. (Cham: Springer International Publishing), 95–98. doi: 10.1007/978-3-319-69185-5_14

[B4] Al-Ghumaiz N. S. Abd-Elmoniem E. M. Motawei M. I. (2017). Salt tolerance and K/Na ratio of some introduced forage grass species under salinity stress in irrigated areas. Commun. Soil Sci. Plant Anal. 48, 1494–1502. doi: 10.1080/00103624.2017.1374398

[B5] Arzani H. Zohdi M. Fish E. Zahedi Amiri G. H. Nikkhah A. Wester D. (2004). Phenological effects on forage quality of five grass species. J. Range Manage. 57 (6), 624–629. doi: 10.2111/1551-5028(2004)057[0624:PEOFQO]2.0.CO;2

[B6] Ashraf M. (2003). Relationships between leaf gas exchange characteristics and growth of differently adapted populations of blue panicgrass (*Panicum antidotale* retz.) under salinity or waterlogging. Plant Sci. 165, 69–75. doi: 10.1016/S0168-9452(03)00128-6

[B7] Assaha D. V. M. Ueda A. Saneoka H. Al-Yahyai R. Yaish M. W. (2017). The role of na+ and k+ transporters in salt stress adaptation in glycophytes. Front. Physiol. 8. doi: 10.3389/fphys.2017.00509 PMC5513949 28769821

[B8] Aziiba E. A. Qiang C. Coulter J. A. (2019). Mechanisms of nitrogen use in maize. Agronomy 9. doi: 10.3390/agronomy9120775

[B9] Bashir E. Y. (1982). Carbohydrate reserves in roots and stem bases of blue panicgrass (*Panicum antidotale*, retz.) as affected by interval and frequency of cutting. [master’s thesis]. (Arizona: University of Arizona).

[B10] Bouras H. Choukr-Allah R. Mosseddaq F. Bouaziz A. Devkota K. P. El Mouttaqi A.. (2022). Does phosphorus fertilization increase biomass production and salinity tolerance of blue panicum (*Panicum antidotale* retz.) in the salt-affected soils of arid regions? Agronomy 12. doi: 10.3390/agronomy12040791

[B11] Butt N. Ahmad M. (1994). Production and management of buffel grass pasture. Progressive Farm. 14, 30–33.

[B12] Cabello P. Agüera E. de la Haba P. (2006). Metabolic changes during natural ageing in sunflower (Helianthus annuus) leaves: expression and activity of glutamine synthetase isoforms are regulated differently during senescence. Physiol. Plantarum 128, 175–185. doi: 10.1111/j.1399-3054.2006.00722.x

[B13] Chen M. Yang Z. Liu J. Zhu T. Wei X. Fan H.. (2018). Adaptation mechanism of salt excluders under saline conditions and its applications. Int. J. Mol. Sci. 19. doi: 10.3390/ijms19113668 PMC6274768 30463331

[B14] Daur I. (2016). Feed value of blue panic (*Panicum antidotale* retz.) grass at different growth stages and under varying levels of humic acid in saline conditions. Turkish J. Field Crops 21, 210–217. doi: 10.17557/tjfc.18296

[B15] Diaz C. Lemaître T. Christ A. Azzopardi M. Kato Y. Sato F.. (2008). Nitrogen recycling and remobilization are differentially controlled by leaf senescence and development stage in arabidopsis under low nitrogen nutrition. Plant Physiol. 147, 1437–1449. doi: 10.1104/pp.108.119040 18467460 PMC2442554

[B16] Elouafi I. Shahid M. A. Begmuratov A. Hirich A. (2020). “The contribution of alternative crops to food security in marginal environments,” in Emerging research in alternative crops. Eds. Hirich A. Choukr-Allah R. Ragab R. (Cham: Springer International Publishing), 1–23. doi: 10.1007/978-3-319-90472-6_1

[B17] Eshghizadeh H. R. Kafi M. Nezami A. (2012). The mechanisms of salinity tolerance in the xero-halophyte blue panicgrass (*Panicum antidotale* retz). Notulae Scientia Biologicae 4 (2), 59–64. doi: 10.15835/nsb427363

[B18] FAO (2018). World livestock: transforming the livestock sector through the sustainable development goals (Rome: Food and Agriculture Organization of the United Nations), 222. doi: 10.4060/ca1201en

[B19] FAO (2022). The state of the world’s land and water resources for food and agriculture – systems at breaking point. main report (Rome, Italy), 362. doi: 10.4060/cb9910en

[B20] Farrag K. Abdelhakim S. G. Abd El-Tawab A. R. Abdelrahman H. (2021). Growth response of blue panic grass (*Panicum antidotale*) to saline water irrigation and compost applications. Water Sci. 35, 31–38. doi: 10.1080/11104929.2020.1860277

[B21] Habib M. Saleem A. Malik A. M. Ahmed S. Arshad S. (2019). Effect of clipping intensity and frequency on growth and morphology of *Panicum antidotale* . Pakistan J. Agric. Res. 32 (4), 642–646. doi: 10.17582/journal.pjar/2019/32.4.642.646

[B22] Hirich A. Choukr-Allah R. Ezzaiar R. Shabbir S. A. Lyamani A. (2021). Introduction of alternative crops as a solution to groundwater and soil salinization in the laayoune area, south Morocco. Euro-Mediterranean J. Environ. Integration 6, 52. doi: 10.1007/s41207-021-00262-7

[B23] Katiyar-Agarwal S. Zhu J. Kim K. Agarwal M. Fu X. Huang A.. (2006). The plasma membrane Na+/H+ antiporter SOS1 interacts with RCD1 and functions in oxidative stress tolerance in arabidopsis. Proc. Natl. Acad. Sci. 103, 18816–18821. doi: 10.1073/pnas.0604711103 17023541 PMC1693745

[B24] Keisham M. Mukherjee S. Bhatla S.C. (2018). Mechanisms of sodium transport in plants-progresses and challenges. Int. J. Mol. Sci. 19. doi: 10.3390/ijms19030647 PMC5877508 29495332

[B25] Khan N. A. Farooq M. W. Ali M. Suleman M. Ahmad N. Sulaiman S. M.. (2015). Effect of species and harvest maturity on the fatty acids profile of tropical forages. JAPS 25, (2015)3.

[B26] Khan N. A. Sulaiman S. M. Hashmi M. S. Rahman S. U. Cone J. W. (2021). Chemical composition, ruminal degradation kinetics, and methane production (*in vitro*) of winter grass species. J. Sci. Food Agric. 101 (1), 179–184. doi: 10.1002/jsfa.10628 32613605

[B27] Kumari S. Chhillar H. Chopra P. Khanna R. R. Khan M. I. R. (2021). Potassium: a track to develop salinity tolerant plants. Plant Physiol. Biochem. 167, 1011–1023. doi: 10.1016/j.plaphy.2021.09.031 34598021

[B28] Madakadze I. C. Stewart K. Peterson P. R. Coulman B. E. Smith D. L. (1999). Switchgrass biomass and chemical composition for biofuel in Eastern Canada. Agron. J. 91, 696–701. doi: 10.2134/agronj1999.914696x

[B29] Maki Y. (1966). Adaptation of blue panicgrass (*Panicum antidotale* Retz.) to warm area of Japan. Japanese J. Grassland Sci. 12, 153–156. doi: 10.14941/grass.12.153

[B30] Masclaux-Daubresse C. Daniel-Vedele F. Dechorgnat J. Chardon F. Gaufichon L. Suzuki A. (2010). Nitrogen uptake, assimilation and remobilization in plants: challenges for sustainable and productive agriculture. Ann. Bot. 105, 1141–1157. doi: 10.1093/aob/mcq028 20299346 PMC2887065

[B31] Mirza S. N. Muhammad N. Qamar I. A. (2002). Effect of growth stages on the yield and quality. Pakistan J. Agric. Res. Vol 17, 145–147.

[B32] Mitchell R. Lee D. K. Casler M. (2014). “Switchgrass,” in Cellulosic energy cropping systems. Ed. Karlen D. L. (Chichester, United Kingdom: John Wiley & Sons), 75–90. doi: 10.1002/9781118676332.ch5

[B33] Mutz J. L. Drawe D. L. (1983). Clipping frequency and fertilization influence herbage yields and crude protein content of 4 grasses in south Texas. J. Range Manag. 36 (5), 582–585. doi: 10.2307/3898345

[B34] Nieves-Cordones M. Alemán F. Martínez V. Rubio F. (2010). The arabidopsis thaliana HAK5 k+ transporter is required for plant growth and k+ acquisition from low k+ solutions under saline conditions. Mol. Plant 3, 326–333. doi: 10.1093/mp/ssp102 20028724

[B35] Núñez-Ramírez R. Sánchez-Barrena M. J. O. Villalta I. Vega J. F. Pardo J. M. Quintero F. J.. (2012). Structural insights on the plant salt-overly-sensitive 1 (SOS1) Na +/H+ antiporter. J. Mol. Biol. 424, 283–294. doi: 10.1016/j.jmb.2012.09.015 23022605

[B36] Oumasst A. Azougay S. Taqarort N. Mimouni A. Hallam J. (2021). Salinity effects on nutrients uptake, biochemical content and growth response of blue panic (*Panicum antidotale* retz) and silage maize (Zea mays l). AFRIMED AJ –Al Awamia 133, 41–62.

[B37] Pequerul A. Pérez C. Madero P. Val J. Monge E. (1993). “A rapid wet digestion method for plant analysis,” in Optimization of plant nutrition: refereed papers from the eighth international colloquium for the optimization of plant nutrition, 31 august – 8 September 1992, Lisbon, Portugal. Eds. Fragoso M. A. C. Van Beusichem M. L. Houwers A. (Netherlands: Dordrecht: Springer), 3–6. doi: 10.1007/978-94-017-2496-8_1

[B38] Pérez P. Morcuende R. Martín Del Molino I. Martínez-Carrasco R. (2005). Diurnal changes of rubisco in response to elevated CO2, temperature and nitrogen in wheat grown under temperature gradient tunnels. Environ. Exp. Bot. 53, 13–27. doi: 10.1016/j.envexpbot.2004.02.008

[B39] R Core Team (2022) R: a language and environment for statistical computing (Vienna, Austria: R Foundation for Statistical Computing). Available at: http://www.R-project.org/ (Accessed April 15, 2023).

[B40] Sarath G. Baird L. M. Mitchell R. B. (2014). Senescence, dormancy and tillering in perennial C4 grasses. Plant Sci. 217–218, 140–151. doi: 10.1016/j.plantsci.2013.12.012 24467906

[B41] Sarwar M. Mahr-un-Nisa Khan M. Mushtaque M. (2006). Chemical composition, herbage yield and nutritive value of *Panicum antidotale* and pennisetum orientale for nili buffaloes at different clipping intervals. Asian-Australas J. Anim. Sci. 19, 176–180. doi: 10.5713/ajas.2006.176

[B42] Shabala S. Cuin T. A. (2008). Potassium transport and plant salt tolerance. Physiol. Plantarum 133, 651–669. doi: 10.1111/j.1399-3054.2007.01008.x 18724408

[B43] Shi H. Quintero F. J. Pardo J. M. Zhu J. K. (2002). The putative plasma membrane NA+/H+ antiporter SOS1 controls long-distance NA+ transport in plants. Plant Cell 14, 465–477. doi: 10.1105/tpc.010371 11884687 PMC152925

[B44] Silveira M. C. T. da, Júnior D. Cunha B. Difante G. Pena K. Silva S.. (2010). Effect of cutting interval and cutting height on morphogenesis and forage accumulation of guinea grass (Panicum maximum). Trop. Grasslands 44, 103–108.

[B45] Sunarpi Horie T. Motoda J. Kubo M. Yang H. Yoda K.. (2005). Enhanced salt tolerance mediated by AtHKT1 transporter-induced na+ unloading from xylem vessels to xylem parenchyma cells. Plant J. 44, 928–938. doi: 10.1111/j.1365-313X.2005.02595.x 16359386

[B46] Van Soest P. J. Robertson J. B. Lewis B. A. (1991). Methods for dietary fiber, neutral detergent fiber, and nonstarch polysaccharides in relation to animal nutrition. J. Dairy Sci. 74, 3583–3597. doi: 10.3168/jds.S0022-0302(91)78551-2 1660498

[B47] Verner D. Treguer D. Redwood J. Christensen J. McDonnell R. Elbert C.. (2018). Climate variability, drought, and drought management in morocco's agricultural sector (Washington, DC: World Bank), 146.

[B48] Virkajärvi P. Saarijärvi K. Rinne M. Saastamoinen M. (2012). “Grass physiology and its relation to nutritive value in feeding horses,” in Forages and grazing in horse nutrition. Eds. Saastamoinen M. Fradinho M. J. Santos A. S. Miraglia N. (Wageningen: Wageningen Academic Publishers), 17–43. doi: 10.3920/978-90-8686-755-4_1

[B49] Walker D. J. Leigh R. A. Miller A. J. (1996). Potassium homeostasis in vacuolate plant cells. Proc. Natl. Acad. Sci. U.S.A. 93, 10510–10514. doi: 10.1073/pnas.93.19.10510 11607707 PMC38416

[B50] Wei T. Simko V. (2021)R package 'corrplot': visualization of a correlation matrix. Available at: https://github.com/taiyun/corrplot (Accessed April 15, 2023).

[B51] Xu Z. Z. Zhou G. S. (2005). Effects of water stress and high nocturnal temperature on photosynthesis and nitrogen level of a perennial grass leymus chinensis, Plant and soil 269, 131–139. doi: 10.1007/s11104-004-0397-y 16685524

[B52] Yang J. Udvardi M. (2018). Senescence and nitrogen use efficiency in perennial grasses for forage and biofuel production. J. Exp. Bot. 69, 855–865. doi: 10.1093/jxb/erx241 29444307

